# Pulmonary expansion manoeuvres compared to usual care on ventilatory mechanics, oxygenation, length of mechanical ventilation and hospital stay, extubation, atelectasis, and mortality of patients in mechanical ventilation: A randomized clinical trial

**DOI:** 10.1371/journal.pone.0295775

**Published:** 2023-12-11

**Authors:** Karina da Silva, Cristino Carneiro Oliveira, Leandro Ferracini Cabral, Carla Malaguti, Anderson José

**Affiliations:** 1 Postgraduate Program in Rehabilitation Sciences and Physical Functional Performance, Federal University of Juiz de Fora, Juiz de Fora, Minas Gerais, Brazil; 2 Department of Cardiorespiratory and Musculoskeletal Physiotherapy, Federal University of Juiz de Fora, Juiz de Fora, Minas Gerais, Brazil; University of Texas Medical Branch at Galveston, UNITED STATES

## Abstract

Pulmonary expansion manoeuvres are therapeutic techniques used to prevent and reverse atelectasis; however, no randomized controlled trials have provided evidence supporting the use of this intervention among individuals on mechanical ventilation. Objective: To evaluate the effects of chest compression-decompression and chest block manoeuvres compared to usual care among patients on mechanical ventilation. Methods: The current study was a randomized clinical trial of adult subjects on mechanical ventilation for 12 to 48 hours. The control group received usual care (passive or active mobilization, manoeuvres for airway clearance and tracheal aspiration). The intervention group received usual care plus two lung expansion manoeuvres, i.e., chest decompression and chest block, while remaining on mechanical ventilation. Assessments were performed before and after usual care, immediately after the intervention and 30 minutes after the intervention. The primary outcome was static compliance. The secondary outcomes were the incidence of atelectasis, dynamic compliance, airway resistance, driving pressure, oxygenation, duration of mechanical ventilation, extubation success, length of hospital and ICU stay, and mortality. Results: Fifty-one participants (67±15 years old, 53% men, 26 in the control group and 25 in the intervention group) were evaluated. No differences in static compliance were observed between groups (intervention minus control) before and after expansion manoeuvres [3.64 ml/cmH_2_O (95% CI: -0.36–7.65, p = 0.074)]. Peripheral oxygen saturation differed between groups before and after expansion manoeuvres, with more favourable outcome observed in the control group [-1.04% (95% CI: -1.94 –-0.14), p = 0.027]. No differences were found in other outcomes. Conclusion: Chest compression-decompression and chest block manoeuvres did not improve ventilatory mechanics, the incidence of atelectasis, oxygenation, the duration of mechanical ventilation, the length of stay in the ICU and hospital, or mortality in individuals on mechanical ventilation. The findings of this study can be valuable for guiding evidence-based clinical practice and developing a therapeutic approach that provides real benefits for this population.

## Introduction

Atelectasis is a common, noninfectious complication in individuals receiving mechanical ventilation. The occurrence of lung collapse leads to changes in ventilatory mechanics, worsens lung injuries, alters the ventilation/perfusion ratio and causes hypoxemia. Lung collapse is also associated with a greater risk of respiratory infections and readmission to or a longer stay in the intensive care unit [1–3].

In this context, lung expansion strategies are adopted to reverse or prevent atelectasis and avoid these complications; these strategies promote an increase in lung volumes by increasing the transpulmonary pressure gradient, which can be caused by an increase in alveolar pressure or a decrease in pleural pressure [4, 5].

The most common methods for promoting lung expansion utilize mechanical ventilation with positive pressure to increase alveolar pressure, such as alveolar recruitment [6], intermittent positive pressure breathing [7], PEEP therapy [7, 8], ventilator hyperinflation [9, 10], and increased inspiratory time [10].

Techniques that utilize an increase in positive pressure present few serious adverse events. Transient hypotension and desaturation are the most common adverse events, but they have been observed to be self-limited and do not lead to severe short-term sequelae. Serious adverse events such as barotrauma and arrhythmias were infrequent [11].

Other techniques for lung expansion induce a reduction in pleural pressure. Passive manual manoeuvres are among these strategies and include compression-decompression [12–17] and chest block techniques [18, 19]. Manual manoeuvres are commonly used in clinical practice in countries with few financial and instrumental resources [20].

However, despite being widely used in clinical practice [20], no randomized controlled trials support the use of passive manual manoeuvres in individuals on mechanical ventilation. Therefore, adequate investigation is necessary to assess whether there are beneficial effects of using manual manoeuvres for lung expansion among individuals under mechanical ventilation. The results of this investigation would guide professionals to adopt an evidence-based practice.

## Objective

This study aimed to compare the effects of usual care plus chest compression-decompression and chest block manoeuvres with the effects of usual care on static compliance among patients undergoing mechanical ventilation. Additionally, the effects of these manoeuvres on ventilatory mechanics, the incidence of atelectasis, oxygenation, mechanical ventilation time, extubation success, the length of ICU stay, the length of hospital stay, and mortality were also evaluated.

## Methods

### Trial design

The study was a parallel, two-group, randomized controlled trial that used concealed allocation with a 1:1 ratio and intention-to-treat analysis designed by independent investigators. The trial was registered at The Brazilian Registry of Clinical Trials (ReBEC) (https://ensaiosclinicos.gov.br, number RBR-7nd6kcb). The Consolidated Standards for Reporting Trials (CONSORT) statement was followed to report the study results [21], and the Template for Intervention Description and Replication (TIDieR) was followed to report the interventions [22]. The study protocol conformed to the ethical guidelines of the Declaration of Helsinki and was approved in November 12, 2019 by the Research Ethics Committee of Santa Casa de Misericórdia de Juiz de Fora/MG (no. 3.701.461). Written informed consent was obtained from all participants.

### Randomization and blinding

The participants included in the study were randomized into two groups. The control group received usual care, and the intervention group received usual care plus lung expansion manoeuvres throughout their duration of mechanical ventilation.

Participants were randomly assigned to each arm of the study at a 1:1 ratio using a list of random numbers generated by an independent individual who was not otherwise involved in the study. The randomization process was conducted through the website www.randomization.com. The allocation was concealed using sequentially numbered, opaque, sealed envelopes. The envelope was opened only after the participant’s enrolment in the study.

The participants were blinded during the ventilatory mechanics and oxygenation assessment because of sedation. The physiotherapists responsible for the interventions for both groups and other care providers were blinded for all outcomes. The evaluator was responsible for the follow-up outcomes was blinded to the participants’ allocation, ventilatory mechanics and oxygenation outcomes. The evaluator who performed the ventilatory mechanics and oxygenation measurements was not blinded to the participants’ allocation.

### Participants

The study was conducted at a single tertiary centre intensive care unit within a general hospital. Participants were recruited between March and October 2021. The inclusion criteria were as follows: all patients hospitalized in a general ICU; aged over 18 years; on mechanical ventilation for a minimum of 12 hours and a maximum of 48 hours. The exclusion criteria were as follows: haemodynamic instability (systolic blood pressure < 90 mmHg or mean blood pressure < 60 mmHg) [23]; osteoarticular lesions in the chest; recent thoracic surgery; pneumothorax; active pulmonary haemorrhage; participation in palliative care programs; transferred to another hospital; inability to measure outcomes due to mechanical ventilator failures. Despite sedation, patients were excluded if it was impossible to measure their respiratory mechanics due to the patient’s ventilatory effort [5, 24, 25].

### Interventions

The interventions were performed in the intensive care unit by physiotherapists from the hospital where the study was conducted. These physiotherapists had undergone prior training by the researchers and were blinded to all outcomes.

#### Control group

Individuals allocated to the control group received the usual care, performed three times daily during the morning, afternoon, and evening periods. Each session of physiotherapy lasted approximately 30 minutes. With the individuals positioned in dorsal decubitus and with the backrest inclined at 30 degrees, passive, active-assisted or active exercises were performed for the core, upper and lower limbs [26], according to the participant’s level of consciousness (assessed by the Richmond Agitation-Sedation Scale (RASS) [27]) and degree of muscle strength (assessed by the Medical Research Council muscle strength scoring system [28]). The increased expiratory flow accelerator technique was performed [29], followed by tracheal aspiration of pulmonary secretions and upper airways, using the open suction circuit, with negative pressure ranging from 80 to 120 mmHg [30]. The FiO_2_ was not elevated to 100% to avoid any impact on oxygenation assessment. Mechanical ventilation was also administered and monitored. The control group did not receive any therapy for lung expansion.

#### Intervention group

In addition to chest compression-decompression and chest block manoeuvres, participants in the intervention group received the same usual care as the control group. These manoeuvres were performed sequentially, in a random order, twice a day, after the usual care, during the morning and afternoon sessions, and throughout the participant’s period of mechanical ventilation. Chest compression-decompression and chest block are passive manual manoeuvres for lung expansion. The rationale behind these manoeuvres is that they improve ventilation by modulating transpulmonary pressure, reducing pleural pressure, and redirecting gas flow to specific lung areas.

#### Pulmonary expansion manoeuvres

*Chest compression-decompression manoeuvre*. The participant was positioned in dorsal decubitus with the backrest inclined at 30 degrees. Soft manual compression was performed on the lower costal region of both thoraxes during the expiratory phase of the ventilatory cycle, followed by a rapid release of the compression at the beginning of the inspiratory phase. The manoeuvre was performed every two ventilation cycles, with a total duration of five minutes [12–17].

*Chest block manoeuvre*. The participant was positioned in dorsal decubitus with the backrest inclined at 30 degrees. Manual compression was performed on the upper and lower regions of one thorax of each participant, and it was continuously maintained for five minutes. Then, the manoeuvre was performed on the other hemithorax [18, 19].

### Assessments

The assessments for measuring ventilatory mechanics and oxygenation were performed on the first day of each participant’s inclusion in the study. These assessments took place at five time points in both groups: before the usual care (A1), 5 minutes after the usual care (A2), 5 minutes after the interventions in the intervention group (or after a 20-minute interval in the control group, comparable to the duration of the interventions) (A3), and 30 minutes after the interventions (A4).

At the end of the hospital stay or death, an evaluator blinded to the participant’s allocation and outcomes of ventilatory mechanics and oxygenation assessed the following outcomes: incidence of atelectasis, length of hospital and ICU stays, duration of mechanical ventilation, extubation success, and in-hospital mortality. Demographic and clinical data, scores on the Charlson Comorbidity Index [31], mechanical ventilation parameters and arterial blood gases on the day of the initial assessment of each participant were also recorded.

### Outcomes

Static compliance of the respiratory system was assessed as the primary outcome. Static compliance of the respiratory system (ml/cmH_2_O) was calculated by dividing the tidal volume by the difference between the plateau pressure and the total positive end-expiratory pressure (PEEPt) [tidal volume/(plateau pressure—PEEPt)].

The secondary outcomes included dynamic compliance (ml/cmH_2_O), which was calculated by dividing the tidal volume by the difference between the peak inspiratory pressure and the PEEPt [tidal volume/(peak inspiratory pressure—PEEPt)]. Airway resistance (cmH_2_O/l/s) was calculated by dividing the difference between the peak inspiratory pressure and the plateau pressure by the inspiratory flow [(peak pressure—plateau pressure)/inspiratory flow]. Driving pressure (cmH_2_O) was calculated by subtracting the PEEPt from the plateau pressure (plateau pressure—PEEPt). Oxygenation was measured by peripheral oxygen saturation.

To measure ventilatory mechanics and oxygenation, the participant was positioned in dorsal decubitus and with the backrest inclined at 30 degrees. Mechanical ventilation was programmed in controlled volume mode, tidal volume set at 6 ml per kilogram of predicted weight, constant inspiratory flow rate and a 3-second inspiratory pause. The evaluator performed the procedures for the measurements, recorded the information presented by monitoring the mechanical ventilator and performed the calculations to evaluate the ventilatory mechanics manually [5, 25]. To minimize error induced by a patient’s respiratory effort during data acquisition, the measurements were repeated until at least five consistent readings were obtained. Waveforms were examined to ensure a flat plateau for reliable measurements [5, 24, 25].

Chest X-rays assessed the incidence of atelectasis and were performed daily on all study participants as part of routine care. All X-rays were reviewed and interpreted by a radiology specialist who was not involved in the study.

Extubation success was defined as the removal of the endotracheal tube and the absence of ventilatory support within 48 hours following extubation [32].

Other secondary outcomes, including mechanical ventilation time, length of ICU and hospital stays, and in-hospital mortality, were assessed based on medical records at the time of hospital discharge or participant death.

### Sample size

The sample size was calculated using G*Power software (Heinrich-Heine-Universität Düsseldorf). When estimating the effect size of 0.865 in a previous study [17], which evaluated changes in lung compliance between the control and intervention groups after the chest compression-decompression manoeuvre, a t test was used with a significance level of 0.05 and a power of 0.80. An allocation ratio of 1:1 (control and intervention) was assumed, and a sample size of 44 participants (22 in each group) was considered adequate. To account for a potential sample loss of 15%, 51 participants were randomized.

### Statistical analysis

Data analysis was performed using IBM Statistical Package for the Social Science (SPSS) software (version 22.0; SPSS Inc., Chicago, IL, USA). The Kolmogorov‒Smirnov and Shapiro‒Wilk tests were conducted to assess the normality of the variables. Continuous variables are presented as either the mean ± standard deviation or median (25–75%), depending on the normality of the data and mean (95% confidence interval). Categorical variables were described using n (%). Static compliance, dynamic compliance, airway resistance, driving pressure and oxygenation were compared within and between groups using generalized linear models, with the baseline value as a covariate, and with minimal significant difference post hoc analysis. These outcomes are expressed as means and confidence intervals (95%). Incidence of atelectasis, length of ICU stay, length of hospital stay, duration of mechanical ventilation, extubation success and in-hospital mortality were compared using the Mann‒Whitney test (for nonnormally distributed variables) or the chi-squared test (for categorical variables). Analyses were conducted according to the intention to treat principle. All tests were 2-sided, and a p value <0.05 was considered statistically significant.

## Results

**Fig 1:**
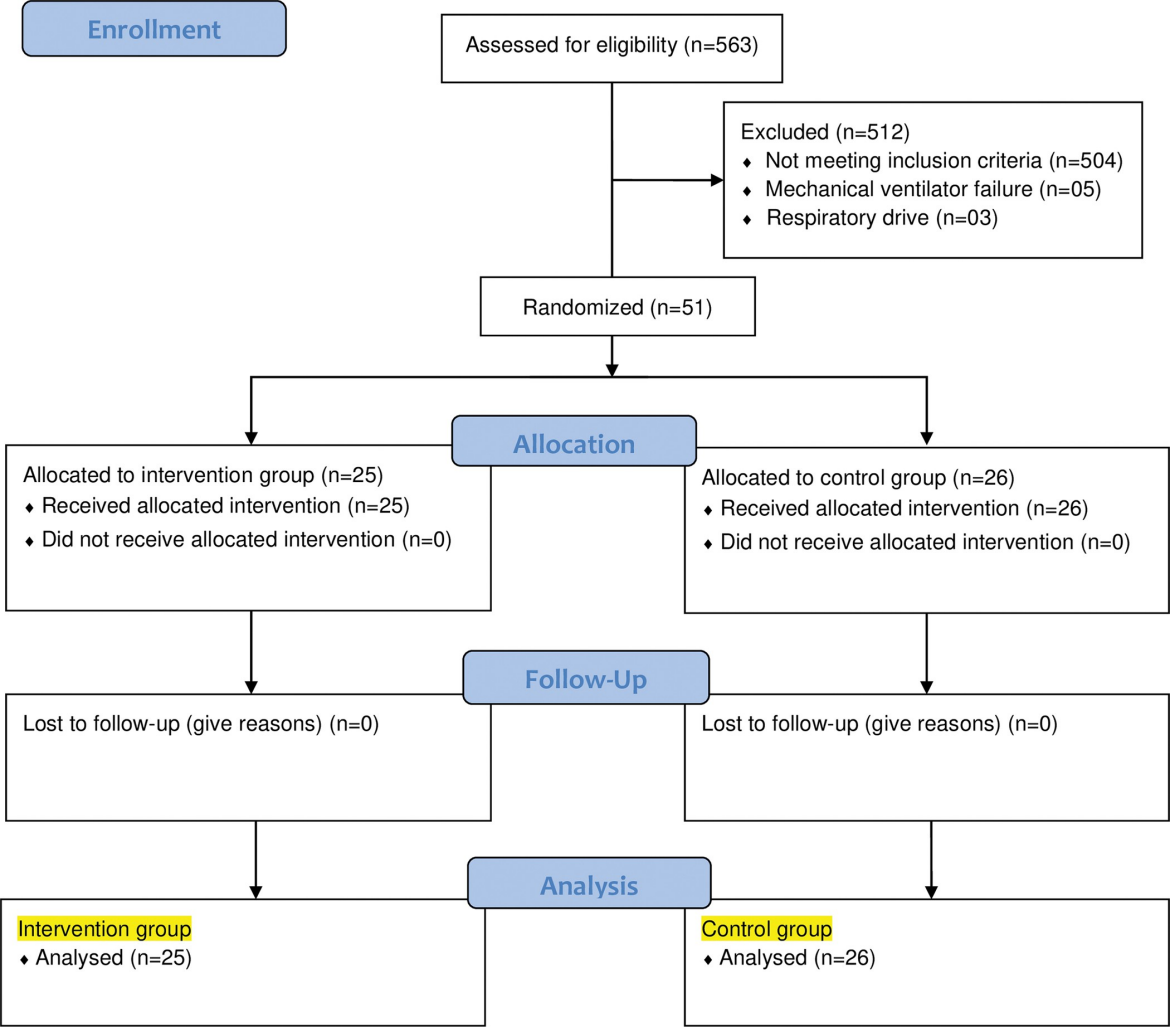
Distribution of participants in the study.

A total of 563 patients were considered eligible for the study. Among them, 512 were excluded (504 did not meet the inclusion criteria; 5 patients were excluded due to mechanical ventilator failure to calculate the ventilatory mechanics; and 3 patients were excluded because they exhibited respiratory effort). The remaining 51 participants were randomly assigned, with 25 in the intervention group and 26 in the control group. These participants received the intended treatment and were analysed for all outcomes (Fig 1). Participants were enrolled between December 2021 and June 2022, and recruitment ended after reaching the sample size.

The mean age of the participants was 67.2 ± 14.9 years, and 52.9% were male. The leading cause of hospitalization was COVID-19 (49%), and the main comorbidity presented by the participants was systemic arterial hypertension (62.7%), while the most commonly used mechanical ventilation mode was volume-controlled ventilation (70.6%) (Table 1). Within-group and between group differences in ventilatory mechanics and oxygenation are shown in Table 2.

**Table 1:** Sample characterization.

	Total (n = 51)	Control group (n = 26)	Intervention group (n = 25)
Age, years	67.2 ± 14.9	63.9 ± 15.5	70.7 ± 13.8
Male gender, n (%)	27 (52.9)	16 (61.5)	11 (44.0)
BMI, kg/m^2^	26.9 ± 6.1	25.9 ± 5.4	27.5 ± 7.0
Diagnosis, n (%)			
COVID-19	25 (49.0)	13 (50.0)	12 (48.0)
Pneumonia	7 (13.7)	5 (19.2)	2 (8.0)
Haemorrhagic stroke	6 (11.8)	2 (7.7)	4 (16.0)
Convulsive crisis	4 (7.8)	2 (7.7)	2 (8.0)
Ischaemic stroke	3 (5.9)	1 (3.8)	2 (8.0)
Others	11 (21.6)	4 (15.4)	7 (28.0)
Comorbidity index, points	3 (2–4)	3 (1–4)	3 (2–4)
Comorbidities, n (%)			
Hypertension	32 (62.7)	17 (65.4)	15 (60.0)
Diabetes Mellitus	18 (35.3)	12 (46.2)	6 (24.0)
Obesity	9 (17.6)	3 (11.5)	6 (24.0)
Thyroid diseases	6 (11.8)	1 (3.8)	5 (20.0)
Previous stroke	6 (11.8)	2 (7.7)	4 (16.0)
Others	32 (62.7)	15 (57.7)	17 (68.0)
Medications, n (%)			
Analgesics/Anaesthetics	48 (94.1%)	26 (100.0%)	22 (88.0%)
Sedatives	44 (86.3%)	22 (84.6%)	22 (88.0%)
Vasoactive	38 (74.5%)	20 (76.9%)	18 (72.0%)
Antibiotics	36 (70.6%)	17 (65.4%)	19 (76.0%)
Anticoagulants	29 (56.9%)	16 (61.5%)	13 (52.0%)
Neuromuscular blockers	22 (43.1%)	10 (38.5%)	12 (48.0%)
Corticosteroids	22 (43.1%)	12 (46.2%)	10 (40.0%)
Mechanical ventilation			
VCV, n (%)	36 (70.6)	18 (69.2)	18 (72.0)
PCV, n (%)	15 (29.4)	8 (30.8)	7 (28.0)
Tidal volume, L	0.386 ± 0.072	0.385 ± 0.077	0.388 ± 0.069
FiO_2_, %	37.96 ± 11.20	40.38 ± 12.88	35.44 ± 8.70
Peak pressure, cmH_2_O	25.69 ± 4.50	25.65 ± 4.42	25.72 ± 4.67
PEEPt, cmH_2_O	9.69 ± 3.67	9.73 ± 3.96	9.64 ± 3.44
Respiratory rate, rpm	23.51 ± 5.84	24.19 ± 6.46	22.80 ± 5.16
Minute volume, L	8.84 ± 2.26	9.19 ± 2.30	8.46 ± 2.21
Arterial blood gas			
pH	7.32 ± 0.09	7.30 ± 0.09	7.36 ± 0.09
PaO_2_, mmHg	106.88 ± 30.66	110.19 ± 30.98	103.44 ± 30.57
PaCO_2_, mmHg	47.29 ± 10.62	48.96 ± 11.40	45.56 ± 9.67
HCO_3_, mEq/L	24.12 ± 5.16	23.77 ± 5.92	24.48 ± 4.32
Base excess	-1.88 ± 5.66	-2.72 ± 6.38	-1.00 ± 4.77
O_2_ sat, %	97 (95–98)	97.5 (95–99)	97 (94.5–98)
PaO_2_/FiO_2_	303.41 ± 122.74	298.60 ± 129.42	308.72 ± 118.12

Data are presented as the mean ± standard deviation, median (25–75% IQR) or absolute numbers (%). BMI = body mass index; VCV: volume-controlled ventilation; PCV: pressure-controlled ventilation: FiO_2_: fraction of inspired oxygen PEEPt: total positive end-expiratory pressure (considering auto-PEEP); pH: hydrogen potential; PaO_2_ = blood pressure of oxygen; PaCO_2_ = blood pressure of carbon dioxide; HCO_3_ = bicarbonate; BE = base excess; O_2_ sat = arterial oxygen saturation.

**Table 2:** Differences within-group and between group (intervention group minus control group) at different evaluation times for ventilatory mechanics and oxygenation.

	Control Group Differences within-group			Intervention Group Differences within-group			Difference between groups (intervention minus control)		
	After usual care (A2) minus baseline (A1)	After rest interval (A3) minus after usual care (A2)	After 30 minutes (A4) minus after rest interval (A3)	After usual care (A2) minus baseline (A1)	After intervention (A3) minus after usual care (A2)	After 30 minutes (A4) minus after intervention (A3)	After usual care (A2) minus baseline (A1)	After intervention (A3) minus after usual care (A2)	After 30 minutes (A4) minus after intervention (A3)
**Static compliance ml/cmH**_**2**_ **O**	1.34 -1.46–4.15	-1.04 -3.85–1.76	2.32 -0.49–5.12	-0.52 -3.38–2.34	2.60 -0.26–5.46	-1.58 -4.44–1.28	-1.87 -5.87–2.14	3.64 -0.36–7.65	-3.89 -7.90–0.11
**Dynamic compliance ml/cmH**_**2**_ **O**	1.59* 0.44–2.73	-0.18 -1.33–0.97	-0.65 -1.80–0.49	0.37 -0.80–1.53	1.09 -0.08–2.26	-1.11 -2.28–0.06	-1.22 -2.86–0.42	1.27 -0.36–0.91	-0.46 -2.10–1.18
**Airway resistance** **ml/cmH**_**2**_ **O/l/s**	0.22 -1.15–1.59	-0.09 -1.46–1.28	-0.38 -1.75–0.99	-1.10 -2.50–0.30	0.05 -1.34–1.45	1.31 -0.09–2.71	-1.32 -3.28–0.64	0.16 -1.82–2.11	1.69 -0.27–3.65
**Driving pressure cmH**_**2**_ **O**	-0.29 -0.81–0.23	0.17 -0.35–0.69	0.02 -0.50–0.53	0.38 -0.14–0.91	-0.42 -0.94–0.11	-0.10 -0.62–0.43	0.68 -0.06–1.42	-0.59 -1.33–0.16	-0.11 -0.85–0.63
**Oxygenation (SpO**_**2**_ **), %**	-0.57 -1.20–0.06	0.27 -0.36–0.90	0.24 -0.39–0.87	-0.73* -1.37 –-0.08	-0.77* -1.41 –-0.12*	0.67* 0.03–1.32	-0.15 -1.06–0.75	-1.04* -1.94 –-0.14	0.43 -0.46–1.34

Data are presented as the mean (95% confidence interval).

* p < 0.05.

No significant difference in the primary outcome (static compliance) was observed between groups. The difference between groups (intervention minus control) before and after expansion manoeuvres was 3.64 (95% CI: -0.36–7.65) ml/cmH_2_O, p = 0.074; effect size: 0.860 (Table 2).

Peripheral oxygen saturation showed difference between groups (intervention minus control) before and after expansion manoeuvres, with more favourable results observed in the control group (-1.04% (95% CI: -1.94 –-0.14), p = 0.024). No other significant between-group differences were observed (Table 2).

On average, the individuals stayed in the ICU for 15 days, stayed in the hospital for 19 days, and spent 11 days on mechanical ventilation. The total in-hospital mortality rate was 72.5%. The incidence of atelectasis was 19% in the control group and 16% in the intervention group. There were no significant between-group differences in the follow-up outcomes (Table 3). No adverse events, significant harms or unintended effects were observed in either group during the application or because of the interventions.

**Table 3:** Follow-up outcomes for the total sample, control and intervention groups.

	Total (n = 51)	Control group (n = 26)	Intervention group (n = 25)	p
Incidence of atelectasis, n (%)	9 (17.6)	5 (19.2)	4 (16)	.762
Length of ICU stay, days	15 (9–30)	16.5 (9.8–30.3)	13 (9–28.5)	.515
Length of hospital stay, days	19 (10–40)	18 (9–41.8)	19 (10–33)	.977
Mechanical ventilation time, days	11 (7–21)	12 (7.5–20.3)	11 (6.5–23)	.880
Extubation, n (%)	20 (39.2)	13 (50)	7 (28)	.108
Extubation success (per extubation), n (%)	15 (75)	10 (76.9)	5 (71.4)	.787
Extubation success (per patient), n (%)	15 (29.4)	10 (38.5)	5 (20)	.787
In-hospital mortality, n (%)	37 (72.5)	16 (61.5)	21 (84.0)	.072

Data are presented as n (%) and median (25–75%).

## Discussion

This study evaluated the effects of chest compression-decompression and chest block manoeuvres compared to usual care in patients receiving mechanical ventilation. Our results revealed that the individuals who received the expansion manoeuvres did not experience improvements in ventilatory mechanics and exhibited worse results in oxygenation when compared to the control group. However, the magnitude of this difference (-1.05%) was negligible since a decrease of 4% or more is considered the desaturation criterion [33, 34]. There were no significant between-group differences in other outcomes studied.

The results found in our study are alarming. A therapeutic approach that is widely used in clinical practice [20] was not shown to be effective. An informal interview was conducted at the hospital where this study was carried out to assess the utilization of the investigated techniques by physiotherapists. The findings revealed that out of the 52 professionals involved in patient care, 43 (77%) reported frequent implementation of these manual therapies for lung expansion. The results of this informal interview are consistent with a study that aimed to investigate the profile of physical therapists working in intensive care units in Brazil [20]. A total of 461 ICUs were investigated in 356 hospital institutions (54.6% private, 43.6% public and 1.8% mixed). This study showed that manual techniques for lung expansion are applied by 99.3% of professionals.

The manual therapies investigated in this study did not cause changes in static lung compliance. Static compliance refers to the change in lung volume for each unit of change in transpulmonary pressure. It is an important indicator for detecting atelectasis [4, 5] and represents the extent to which the lung can accommodate the air volume. Substantial changes in transpulmonary pressure are needed to recruit collapsing lung units and increase static lung compliance in individuals on mechanical ventilation. However, these changes were not achieved with the manual manoeuvres studied.

Several studies have assessed manual manoeuvres’ effectiveness for lung expansion [12–19]. However, it is essential to highlight the low methodological quality and the limited outcomes investigated in these studies. These studies have a cross-sectional or case report design, which needs to be improved to evaluate the effectiveness of therapeutic interventions. Additionally, they did not have control groups, utilized convenience samples with small sample sizes, examined outcomes that needed to be more relevant for assessing lung expansion or the effectiveness of the manoeuvres, and needed to consider the participants’ progression during hospitalization.

Our study also found no significant differences between groups in the incidence of atelectasis, length of mechanical ventilation, length of ICU and hospital stay, extubation rate and success, or in-hospital mortality. As expected, since ventilatory mechanics and oxygenation did not show any benefits from applying expansion manoeuvres, the clinical outcomes that these factors could have influenced the improvement also remained unchanged.

Our study makes an important contribution to clinical practice by demonstrating the lack of benefits in adopting compression-decompression and chest block manoeuvres during therapy for hospitalized individuals undergoing mechanical ventilation. This provides an opportunity for applying or optimizing other therapeutic strategies that are known to be effective. Evidence-based practice needs to be adopted in managing these patients so that there is a real benefit from a therapeutic intervention.

Another strength of this study is its external validity. The inclusion criteria were comprehensive, including a diverse sample of individuals with different diagnoses, severity levels, and clinical conditions, thus simulating a real-life situation encountered by physiotherapists in their clinical practice. Therefore, the study provides a basis for therapeutic decision-making and interventions.

This study has some limitations. The evaluator who performed the ventilatory mechanics and oxygenation measurements was not blinded to the participants’ allocation. We attempted to mitigate this limitation by recruiting individuals who were blinded to the evaluation of other study outcomes. In clinical practice, the studied manoeuvres exhibit variability in their execution and application time. Since there is no consensus or guideline on the techniques, these findings may be subject to modification due to methodological differences. The interventions were administered by different physiotherapists, which could introduce variability in the techniques employed. However, the team responsible for conducting the interventions received training and continuous supervision from the researchers to mitigate this potential effect. The sample did not include patients with preexisting atelectasis. However, one of the indications for lung expansion manoeuvres is the prevention of atelectasis [13, 15, 16]. Additionally, the study was designed to simulate clinical practice by performing lung expansion manoeuvres to prevent lung collapse in all patients on mechanical ventilation, even without an atelectasis diagnosis. In clinical practice, these manoeuvres are commonly performed in all individuals on mechanical ventilation; therefore, the study simulated a real clinical situation. We suggest that future studies focus specifically on patients with atelectasis. Finally, since the manoeuvres in our study were performed together, it is impossible to isolate each technique’s individual effects. Further studies are warranted to evaluate the individual effects of these techniques separately.

After the research ethics committee approved the research project, the following changes were made to the study: the participant recruitment date was adjusted from October 2019 and February 2020 to March and October 2021 due to delays in obtaining approval from the administrative authorities and the ethics committee. Static compliance is a variable directly related to the response to lung expansion, so it was maintained as the primary outcome, while the other outcomes were classified as secondary. Driving pressure was included as an outcome due to its relevance in assessing lung expansion. Tidal and minute volume outcomes were removed because many patients were receiving mechanical ventilation via the volume-controlled mode. Therefore, no changes in ventilation would be observed in these individuals after the interventions. The appropriate number of participants for the study was revised from 34 to 44 due to an error in the sample size calculation. It was discovered that the effect size in the study conducted by Unoki et al. [17] was 0.865 instead of 1.0. The analysis of some outcomes was modified based on the subsequent observation that the more appropriate analysis for comparing two groups over time in four assessments is generalized linear models instead of Student’s t test or the Mann‒Whitney test. As these methodological changes did not involve any modifications to the interventions, they were communicated and approved by The Brazilian Registry of Clinical Trials (ReBEC).

## Conclusion

Compared to usual care, pulmonary expansion manoeuvres (chest compression-decompression and chest block) may not improve the ventilatory mechanics, incidence of atelectasis, oxygenation, time on mechanical ventilation, extubation success, duration of ICU and hospital stays, and in-hospital mortality among individuals on mechanical ventilation. The results of this study can be used to guide evidence-based clinical practice that provide tangible benefits to this population. Further research is needed to confirm the findings of this study.

## Supporting information

S1 Data Data file anonymized. (XLSX)

S1 File (DOCX)
