# Supplementary material for: Pulmonary expansion manoeuvres compared to usual care on ventilatory mechanics, oxygenation, length of mechanical ventilation and hospital stay, extubation, atelectasis, and mortality of patients in mechanical ventilation: A randomized clinical trial
Source: PLoS One. 2023 Dec 11;18(12):e0295775. doi: 10.1371/journal.pone.0295775 (PMC10712844; doi:10.1371/journal.pone.0295775)
Supplement: S1 File — (DOCX) [file pone.0295775.s002.docx]

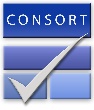


CONSORT 2010 checklist of information to include when reporting a randomised trial*

| Section/Topic | Item No | Checklist item | Reported on page No |
| --- | --- | --- | --- |
| Title and abstract | | | |
|  | 1a | Identification as a randomised trial in the title | Page 01 (Title page) |
|  | 1b | Structured summary of trial design, methods, results, and conclusions (for specific guidance see CONSORT for abstracts) | Page 02 |
| Introduction | | | |
| Background and objectives | 2a | Scientific background and explanation of rationale | Page 04 |
|  | 2b | Specific objectives or hypotheses | Page 05 |
| Methods | | | |
| Trial design | 3a | Description of trial design (such as parallel, factorial) including allocation ratio | Page 05 |
|  | 3b | Important changes to methods after trial commencement (such as eligibility criteria), with reasons | Page 19 |
| Participants | 4a | Eligibility criteria for participants | Page 06 |
|  | 4b | Settings and locations where the data were collected | Page 06 |
| Interventions | 5 | The interventions for each group with sufficient details to allow replication, including how and when they were actually administered | Pages 07-08 |
| Outcomes | 6a | Completely defined pre-specified primary and secondary outcome measures, including how and when they were assessed | Pages 09-10 |
|  | 6b | Any changes to trial outcomes after the trial commenced, with reasons | Page 19 |
| Sample size | 7a | How sample size was determined | Page 10 |
|  | 7b | When applicable, explanation of any interim analyses and stopping guidelines | N/A |
| Randomisation: |  |  |  |
| Sequence generation | 8a | Method used to generate the random allocation sequence | Page 06 |
|  | 8b | Type of randomisation; details of any restriction (such as blocking and block size) | Page 06 |
| Allocation concealment mechanism | 9 | Mechanism used to implement the random allocation sequence (such as sequentially numbered containers), describing any steps taken to conceal the sequence until interventions were assigned | Page 06 |
| Implementation | 10 | Who generated the random allocation sequence, who enrolled participants, and who assigned participants to interventions | Page 06 |
| Blinding | 11a | If done, who was blinded after assignment to interventions (for example, participants, care providers, those assessing outcomes) and how | Page 06 |
|  | 11b | If relevant, description of the similarity of interventions | N/A |
| Statistical methods | 12a | Statistical methods used to compare groups for primary and secondary outcomes | Pages 11 |
|  | 12b | Methods for additional analyses, such as subgroup analyses and adjusted analyses | N/A |
| Results | | | |
| Participant flow (a diagram is strongly recommended) | 13a | For each group, the numbers of participants who were randomly assigned, received intended treatment, and were analyzed for the primary outcome | Flow diagram and page 11 |
|  | 13b | For each group, losses and exclusions after randomisation, together with reasons | N/A |
| Recruitment | 14a | Dates defining the periods of recruitment and follow-up | Page 06 |
|  | 14b | Why the trial ended or was stopped | Page 06 |
| Baseline data | 15 | A table showing baseline demographic and clinical characteristics for each group | Table 1 |
| Numbers analyzed | 16 | For each group, number of participants (denominator) included in each analysis and whether the analysis was by original assigned groups | Page 12-16 and tables 2, 3, and 4 |
| Outcomes and estimation | 17a | For each primary and secondary outcome, results for each group, and the estimated effect size and its precision (such as 95% confidence interval) | Pages 13-16 and tables 2, 3, and 4 |
|  | 17b | For binary outcomes, presentation of both absolute and relative effect sizes is recommended | Page 15-16 and Table 4 |
| Ancillary analyses | 18 | Results of any other analyses performed, including subgroup analyses and adjusted analyses, distinguishing pre-specified from exploratory | N/A |
| Harms | 19 | All-important harms or unintended effects in each group (for specific guidance see CONSORT for harms) | Pages 13-16 |
| Discussion | | | |
| Limitations | 20 | Trial limitations, addressing sources of potential bias, imprecision, and, if relevant, multiplicity of analyses | Page 18-19 |
| Generalizability | 21 | Generalizability (external validity, applicability) of the trial findings | Page 18 |
| Interpretation | 22 | Interpretation consistent with results, balancing benefits and harms, and considering other relevant evidence | Pages 16-18 |
| Other information | | |  |
| Registration | 23 | Registration number and name of trial registry | Page 03 |
| Protocol | 24 | Where the full trial protocol can be accessed, if available | N/A |
| Funding | 25 | Sources of funding and other support (such as supply of drugs), role of funders | N/A |

*We strongly recommend reading this statement in conjunction with the CONSORT 2010 Explanation and Elaboration for important clarifications on all the items. If relevant, we also recommend reading CONSORT extensions for cluster randomised trials, non-inferiority and equivalence trials, non-pharmacological treatments, herbal interventions, and pragmatic trials. Additional extensions are forthcoming: for those and for up-to-date references relevant to this checklist, see [www.consort-statement.org](http://www.consort-statement.org).
